# p11 in Cholinergic Interneurons of the Nucleus Accumbens Is Essential for Dopamine Responses to Rewarding Stimuli

**DOI:** 10.1523/ENEURO.0332-18.2018

**Published:** 2018-11-08

**Authors:** Y. Hanada, Y. Kawahara, Y. N. Ohnishi, T. Shuto, M. Kuroiwa, N. Sotogaku, P. Greengard, Y. Sagi, A. Nishi

**Affiliations:** 1 Department of Pharmacology, Kurume University School of Medicine, Kurume, Fukuoka 830-0011, Japan,; 2 Laboratory of Molecular and Cellular Neuroscience, the Rockefeller University, New York, NY 10065

**Keywords:** acetylcholine, anhedonia, cholinergic interneuron, dopamine, nucleus accumbens, p11

## Abstract

A recent study showed that p11 expressed in cholinergic interneurons (CINs) of the nucleus accumbens (NAc) is a key regulator of depression-like behaviors. Dopaminergic neurons projecting to the NAc are responsible for reward-related behaviors, and their function is impaired in depression. The present study investigated the role of p11 in NAc CINs in dopamine responses to rewarding stimuli. The extracellular dopamine and acetylcholine (ACh) levels in the NAc were determined in freely moving male mice using *in vivo* microdialysis. Rewarding stimuli (cocaine, palatable food, and female mouse encounter) induced an increase in dopamine efflux in the NAc of wild-type (WT) mice. The dopamine responses were attenuated (cocaine) or abolished (food and female mouse encounter) in constitutive p11 knock-out (KO) mice. The dopamine response to cocaine was accompanied by an increase in ACh NAc efflux, whereas the attenuated dopamine response to cocaine in p11 KO mice was restored by activation of nicotinic or muscarinic ACh receptors in the NAc. Dopamine responses to rewarding stimuli and ACh release in the NAc were attenuated in mice with deletion of p11 from cholinergic neurons (ChAT-p11 cKO mice), whereas gene delivery of p11 to CINs restored the dopamine responses. Furthermore, chemogenetic studies revealed that p11 is required for activation of CINs in response to rewarding stimuli. Thus, p11 in NAc CINs plays a critical role in activating these neurons to mediate dopamine responses to rewarding stimuli. The dysregulation of mesolimbic dopamine system by dysfunction of p11 in NAc CINs may be involved in pathogenesis of depressive states.

## Visual Abstract

**Figure:**
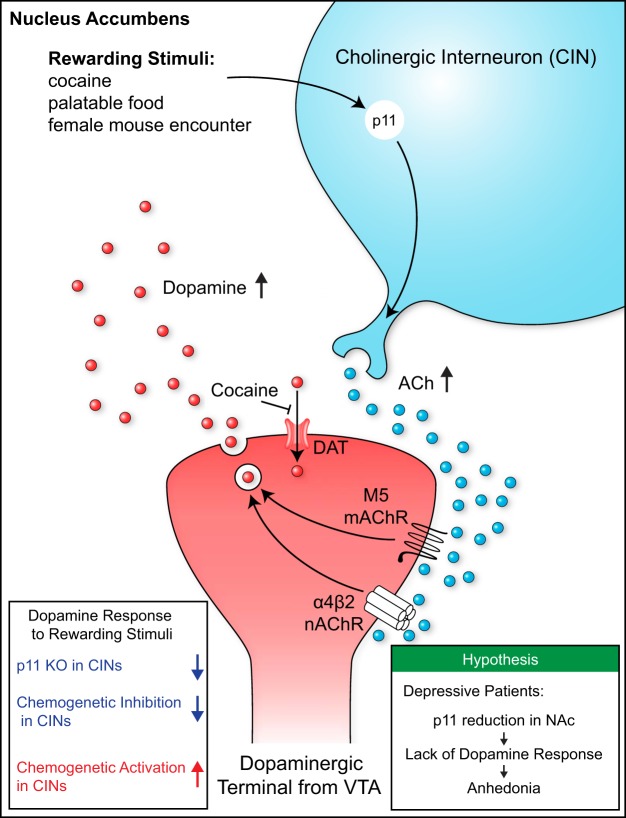

## Significance Statement

p11 is a critical regulator of cholinergic interneuron (CIN) activity as measured by the dopamine response of the mesolimbic dopamine pathway to rewarding stimuli. p11 is required for reward-mediated nucleus accumbens (NAc) CIN activation and induction in acetylcholine (ACh) release, resulting in the enhancement of dopamine release. The reduction of p11 expression in NAc CINs is tightly associated with anhedonia as well as other depression-like symptoms of behavioral despair. To improve therapeutic efficacy of antidepressants for anhedonia, a new type of antidepressant directly or indirectly acting on the mesolimbic dopamine pathway needs to be developed. For this purpose, therapeutic strategies that increase the function of p11 and its signaling pathway in NAc CINs may have an impact on antidepressant efficacy.

## Introduction

Depressive patients show a variety of mood-related symptoms: increased negative affect (e.g., depressed mood, guilt, anxiety) and decreased positive affect [e.g., anhedonia (loss of interest or pleasure), decreased motivation; Clark and Watson, 1991]. Although antidepressants, which upregulate serotonin and/or noradrenaline neurotransmission, effectively alleviate negative affect, they are relatively ineffective at improving positive affect (Shelton and Tomarken, 2001; Craske et al., 2016). The ineffectiveness can be explained by the fact that anhedonia is associated with a deficit in the dopamine reward circuit (Der-Avakian and Markou, 2012; Russo and Nestler, 2013). Since anhedonia is a predictor of poor long-term outcomes including poor treatment response and suicide (Craske et al., 2016), further understanding of the neurobiology of anhedonia in depression is required to improve therapeutic efficacy of current antidepressant treatments. p11 (S100A10) is a member of the S100 EF-hand protein family, and is known to play pivotal roles in the pathophysiology of depression (Svenningsson et al., 2006, 2013). Extensive studies on the function of p11 revealed that p11 potentiates serotonin neurotransmission via multiple mechanisms including recruitment of 5-HT_1B_ and 5-HT_4_ receptors at the cell surface (Svenningsson et al., 2006; Warner-Schmidt et al., 2009), and regulates depression-like behaviors and responses to antidepressants (Svenningsson et al., 2013; Medrihan et al., 2017). Constitutive p11 knock-out (KO) mice show depression-like behaviors, including increased behavioral despair and anhedonia (Svenningsson et al., 2006; Warner-Schmidt et al., 2009; Alexander et al., 2010). p11 is expressed in various brain regions (Milosevic et al., 2017), and p11 expressed in the nucleus accumbens (NAc; Alexander et al., 2010; Warner-Schmidt et al., 2012), cerebral cortex (Schmidt et al., 2012; Seo et al., 2017b), hippocampus (Egeland et al., 2010; Oh et al., 2013; Medrihan et al., 2017), and habenula (Seo et al., 2017a) affects depression-like behaviors via a variety of neural mechanisms. Furthermore, in depressed patients, the expression of p11 is reduced in the anterior cingulate cortex and NAc (Svenningsson et al., 2006; Alexander et al., 2010).

The NAc receives dopaminergic input from the ventral tegmental area (VTA) and has been implicated as a key brain region of the reward system (Russo and Nestler, 2013; Hu, 2016). In the NAc, p11 is expressed in a cell type-specific manner: low levels in medium spiny neurons (MSNs) and high levels in cholinergic interneurons (CINs; 30-fold higher than non-cholinergic neurons; Warner-Schmidt et al., 2012). p11 in CINs has been shown to be a key regulator of depression-like behavior: (1) mice with p11 knock-down in NAc show depression-like behaviors (Alexander et al., 2010) and (2) p11 KO mice in choline acetyltransferase (ChAT) cells (ChAT-p11 cKO mice) show depression-like behaviors and the behaviors are rescued by overexpression of p11 in NAc CINs (Warner-Schmidt et al., 2012).

Cholinergic tone in the mesolimbic dopamine system plays an important role in behavioral responses to psychostimulants and natural reward (Hoebel et al., 2007; Williams and Adinoff, 2008). In the NAc, psychostimulants increase the activity of CINs (Berlanga et al., 2003; Witten et al., 2010) and acetylcholine (ACh) release (Consolo et al., 1999). Feeding induces a gradual increase in ACh, which is known to have a role in the onset of satiation (Hoebel et al., 2007). Effects of cholinergic neurotransmission on responses to psychostimulants and natural reward-related behaviors are highly dependent on physiologic and experimental conditions (Consolo et al., 1999; Gonzales and Smith, 2015) and contradictory (Hikida et al., 2001; Hoebel et al., 2007; Witten et al., 2010; Grasing, 2016). Grasing (2016) proposed a threshold model to explain the inverted U-shape dose-response of ACh, in which moderate activation of CINs increases the reward probability, whereas activation of CINs above a certain threshold reduces it.p11 KO mice show altered cocaine conditional place preference (CPP; Arango-Lievano et al., 2014; Thanos et al., 2016), suggesting that p11 plays a pivotal role in the regulation of reward. However, a role for p11 in NAc dopamine neurotransmission has not been established. Therefore, we investigated the role of p11 in dopamine neurotransmission in the NAc and prefrontal cortex (PFC) after exposing mice to cocaine or to natural rewards. The present study demonstrates that p11 is required to activate CINs to increase ACh release in response to rewarding stimuli in the NAc, leading to activation of the mesolimbic (VTA-NAc) dopamine system.

## Materials and Methods

### Animals

Male constitutive p11 KO (Svenningsson et al., 2006), ChAT-Cre (GENSAT, GM60) and ChAT-p11 cKO (Warner-Schmidt et al., 2012) mice at 8–12 weeks of age were used. ChAT-p11 cKO mice were generated by breeding floxed p11 mice with ChAT-Cre mice (Warner-Schmidt et al., 2012). Mice were housed two to five per cage and maintained on a 12/12 h light/dark cycle (lights on from 7 A.M. to 7 P.M.) with access to standard mouse chow and water ad libitum. All mice used in this study were handled in accordance with the Guide for the Care and Use of Laboratory Animals as adopted by the National Institutes of Health, and the specific protocols were approved by the Institutional Animal Care and Use Committee. All efforts were made to minimize the number of animals used.

### Drugs

Cocaine (Takeda Pharmaceutical Companies), nicotine (Sigma-Aldrich), oxotremorine (Sigma-Aldrich), dihydro-β-erythroidin (DHβE; Sigma-Aldrich), atropine (Sigma-Aldrich), and clozapine N-oxide (CNO; Cayman Chemical) were dissolved in Ringer’s solution for local infusion.

### Surgery and brain dialysis

**Figure 1.**
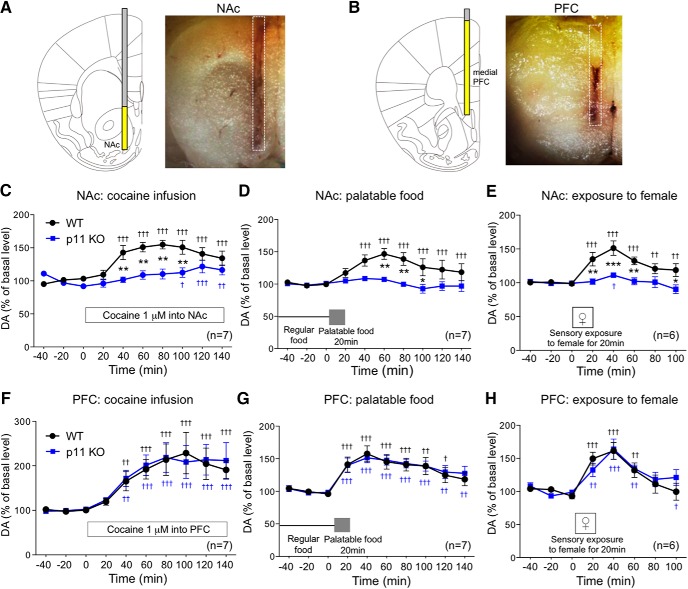
The dopamine (DA) response to rewarding stimuli in the NAc and PFC of constitutive p11 KO mice. ***A***, ***B***, Representative location of a microdialysis probe placed in the mouse NAc (***A***) and PFC (***B***) (Paxinos and Franklin, 2001). The position of dialysis membrane is indicated with yellow color. ***C–H***, The effects of cocaine infusion (1 µM) into the NAc (***C***) or PFC (***F***), exposure to palatable food (***D***, ***G***), and exposure to female mice (***E***, ***H***) on the extracellular levels of DA in the NAc (***C–E***) and PFC (***F–H***) of WT and constitutive p11 KO mice. The DA levels were determined with *in vivo* microdialysis. The basal values for each group were obtained as the average of three stable baseline samples, and all values are calculated as a percentage of the basal values within the same group (100%). Data represent mean ± SEM. *p < 0.05, **p < 0.01, ***p < 0.001 versus WT mice; two-way ANOVA and Bonferroni multiple comparison test. ^†^*p* < 0.05, ^††^*p* < 0.01, ^†††^*p* < 0.001 versus the basal levels of DA in the same group. The number of mice is indicated in parentheses.

Microdialysis was performed with an I-shaped cannula. Microdialysis probes were implanted in the unilateral NAc (exposed length 1.5 mm) or PFC (exposed length, 3.5 mm) of 12-week-old mice under pentobarbital (50 mg/kg, i.p.) and xylazine (8 mg/kg, i.p.) anesthesia and local application of 10% lidocaine. The coordinates of the implantation into the NAc were A/P +1.4 mm, L/M 0.6 mm from the bregma, and V/D 4.5 mm from the dura at an angle of 0° in the coronal plane (Fig. 1*A*). The coordinates of the implantation into the PFC were A/P + 1.9 mm, L/M 0.3 mm from the bregma, and V/D 2.8 mm from the dura at an angle of 0° in the coronal plane (Fig. 1*B*). After surgery, the mice were housed individually in plastic cages (30 × 30 × 40 cm).

Microdialysis experiments were conducted 24–48 h after implantation of the probe, as previously described (Kaneko et al., 2016). An on-line approach for real-time quantification of dopamine was used, in which the probes were perfused with Ringer’s solution at a flow rate of 2.0 µl/min. The 20-min sample fractions collected through dialysis probes were directly injected to HPLC using a reverse-phase column (150 × 4.6 mm; Supelco LC18) with electrochemical detection. An EP-300 pump (EICOM) was used in conjunction with an electrochemical detector (potential of the first cell, +180 mV; potential of the second cell, -180 mV; ESA). The mobile phase was a mixture of 4.1 g/l sodium acetate adjusted to pH 5.5, 50 mg/l Na_2_EDTA, 140 mg/l octanesulfonic acid, and 10% methanol. The flow rate was 0.4 ml/min. The detection limit of assay was ∼0.3 fmol per sample (on-column). The composition of the Ringer’s solution was: 140.0 mM NaCl, 4.0 mM KCl, 1.2 mM CaCl_2_, and 1.0 mM MgCl_2_. At the end of the experiment, the mice were given an overdose of sevoflurane and brains were fixed with 4% paraformaldehyde via intracardiac infusion. Coronal sections (50 µm) were cut, and dialysis probe placement was localized using the atlas of Paxinos and Franklin (Paxinos and Franklin, 2001) as reference. Mice in which dialysis probes were misplaced, were not included in data analysis.

For analysis of ACh, the microdialysis probes were perfused with Ringer’s solution at a flow rate of 1.0 µl/min. The 10-min dialysate fractions were collected, and ACh content was detected using HPLC-ECD system with AC-GEL separation column (2.0 ID × 150 mm) with a platinum working electrode (Eicom-USA) as previously reported (Virk et al., 2016). ACh content in each dialysate sample was determined using subsequent standards with known amounts of ACh. The threshold for detection was 2.44 fmol/min ACh. Neostigmine (100 nM) was added to the dialysis solution to establish continuous ACh efflux.

### ChAT cell-specific expression of p11, rM4D(Gi-DREADD), and rM3D(Gs-DREADD) using AAV vectors

For overexpression of p11 in ChAT cells of the NAc in ChAT-p11 cKO mice, *AAV-loxP-RFP/stop-loxP-p11* (2.7 × 10^12^ virus molecules/ml) and its control vector, *AAV-loxP-RFP/stop-loxP-YFP* (5.2 × 10^12^ virus molecules/ml), were used (Warner-Schmidt et al., 2012). RFP was expressed in Cre recombinase-negative cells such as MSNs, and p11 or YFP was expressed in ChAT cells of the NAc, where the Cre recombinase was expressed under control of ChAT promoter.

For chemogenetic modulation of ChAT cell functions, *rAAV2/hsyn-DIO-rM3D(Gs)-mCherry* (6.6 × 10^12^ virus molecules/ml), *rAAV2/hsyn-DIO-rM4D(Gi)-mCherry* (3.7 × 10^12^ virus molecules/ml) and its control vector, *rAAV2/Ef1a-DIO-mCherry* (3.2 × 10^12^ virus molecules/ml), purchased from University of North Carolina (UNC) Vector Core, were used.

Viruses were infused bilaterally into the NAc in ChAT-p11 cKO mice at eight weeks old, under pentobarbital (50 mg/kg, i.p.) and xylazine (8 mg/kg, i.p.) anesthesia and local application of 10% lidocaine. The coordinates of the infusions into the NAc were A/P +1.4 mm, L/M ±0.6 mm from the bregma, and V/D 3.7 mm from the dura at an angle of 0° in the coronal plane. All infusions were performed using a 5-μl Hamilton syringe with a 33-G needle attached at a rate of 0.1 μl/min. To prevent reflux after infusion, the injection needle was left in the place for 15 min. The needle was withdrawn a short distance (0.3 mm) every 3 min, and this procedure was repeated until the needle was completely removed. Four weeks later, the microdialysis probe was implanted, and *in vivo* microdialysis assessments were performed.

### Rewarding stimuli

#### Cocaine infusion

Cocaine infusion at 1 μM into the NAc induced the increase of extracellular dopamine (150–200% of basal level), which is similar to the increase of dopamine in the NAc induced by systemic cocaine administration (at low to moderate doses) with rewarding effects (Brown et al., 1991; Tourino et al., 2012). During the experimental period, cocaine at 1 μM was infused into the NAc or PFC through the dialysis membrane for 140 min after obtaining three stable consecutive samples of dopamine differing by <10%.

#### Palatable food

After microdialysis probe implantation, flavored cereal food (Asahi Food & Health Care Co.), to which mice exhibit palatability, was introduced to the mice in the acrylic box 24 h before the start of the experiment to promote habituation (Kawahara et al., 2013). Flavored cereal food was removed 1 h before the start of experiments on the day of the experiments, whereas mice had free access to regular food. During the experimental period, after obtaining three stable consecutive samples of dopamine, regular food was removed and then mice were exposed to palatable food for 20 min.

#### Exposure to a female mouse

During the experimental period, male mice were exposed to female C57BL/6N mice at the same age, purchased from Japan SLC, after obtaining three stable consecutive samples of dopamine. Female mice enclosed in a clear acrylic cage (10 × 10 × 20 cm) with 1 cm slits were placed in the plastic cage (30 × 30 × 40 cm) of male mouse for 20 min, and thereafter, the female mouse and the clear acrylic cage were removed.

### Immunohistochemistry

Mice were deeply anesthetized with sodium pentobarbital and were transcardially perfused with 4% paraformaldehyde in phosphate buffer (0.1 M; pH 7.4). Three to four hours after perfusion, the brains were removed and further fixed with 4% paraformaldehyde overnight at 4°C. Coronal sections of the NAc (50 μm in thickness) were cut with a vibrating blade microtome (VT1000S, Leica Microsystems). Sections were processed for immunohistochemistry using the free-floating method, as described previously (Fukuda et al., 1996). Sections were incubated with a goat anti-p11 (S100A10) antibody (catalog #AF2377, RRID:AB_2183469; 1:200 dilution; R&D Systems) or a goat anti-ChAT antibody (catalog #AB144P, RRID:AB_2079751; 1:500 dilution; Millipore) for one week at 20°C. Antibody binding was visualized with Alexa Fluor 488- or 647-conjugated donkey anti-goat IgG (1:800 dilution; Jackson ImmunoResearch). Sections were mounted using antifade media (Vectashield; Vector Laboratories) and examined with a confocal laser-scanning microscope, LSM 5 PASCAL (Zeiss) or FV-1000 (Olympus).

### Statistical analysis

The data are displayed as the mean ± SEM. For analyses of microdialysis data, all values were expressed as a percentage of the basal values (100%) for each group, obtained as the average of three and six stable baseline samples for dopamine and ACh, respectively. The values obtained after rewarding stimuli were compared with the basal values using mixed linear models with time as a covariate, and Bonferroni’s correction was applied for multiple comparisons using the SAS MIMED procedure (version 9.4, SAS Institute). Repeated measures two-way ANOVA were used to compare the experimental groups (JMP Pro, SAS Institute). The basal values of dopamine and/or its metabolites were compared with unpaired Student’s *t* test (Table 1), and the effects of CNO on dopamine levels in ChAT-p11 cKO mice with Gs DEADD viral injection were compared with one-way ANOVA followed by Newman–Keuls *post hoc* test. The analyses were performed using Prism 5.0 software (GraphPad); *p* < 0.05 was considered to be significant. Details of the statistical analysis are listed in Table 2.

**Table 1.** Basal levels of dopamine, dopamine metabolites, and ACh

Mouse	Brain region	DA	DOPAC	HVA	ACh
(fmol/sample)	(pmol/sample)	(pmol/sample)	(fmol/sample)
WT	NAc	41.28 ± 5.47 (22)	5.725 ± 0.592 (21)	12.73 ± 2.49 (15)	nd
p11 KO (constitutive p11 KO)	NAc	42.35 ± 6.31 (17)	5.777 ± 0.917 (16)	13.57 ± 2.47 (9)	nd
WT	PFC	12.00 ± 4.44 (8)	0.894 ± 0.121 (7)	4.863 ± 1.447 (3)	nd
p11 KO (constitutive p11 KO)	PFC	6.35 ± 1.77 (7)	1.020 ± 0.142 (7)	5.143 ± 0.743 (4)	nd
WT (ChAT-cre^-/-^ P11^flox/flox^)	NAc	46.72 ± 9.94 (8)	6.549 ± 1.264 (8)	nd	428.7 ± 75.65 (12)
ChAT p11 cKO (ChAT-cre P11^flox/flox^)	NAc	64.13 ± 11.63 (8)	6.485 ± 0.857 (8)	nd	557.0 ± 116.6 (8)
ChAT p11 cKO + *AAV-YFP*	NAc	29.19 ± 6.45 (8)	nd	nd	nd
ChAT p11 cKO + *AAV-p11*	NAc	35.35 ± 10.08 (8)	nd	nd	nd
ChAT p11 cKO + *AAV-mCherry*	NAc	54.18 ± 21.21 (6)	nd	nd	nd
ChAT p11 cKO + *AAV-rM3D (Gs)*	NAc	50.77 ± 22.85 (6)	nd	nd	nd
ChAT p11 cKO + *AAV-mCherry*	NAc	117.8 ± 37.19 (8)	nd	nd	nd
ChAT p11 cKO + *AAV-hM4D (Gi)*	NAc	77.90 ± 21.02 (13)	nd	nd	nd

Data represent mean ± SEM. The numbers of experiments are shown in the parentheses.

DA, dopamine; DOPAC, 3,4-dihydroxyphenylacetic acid; HVA, homovanillic acid; ACh, acetylcholine; nd, not determined.

**Table 2.** Statistical analyses for data

Set of data		Type of statistical analysis	Results of statistical analysis	
Figure 1				
*C*: DA levels in the NAc with cocaine infusion into the NAc				
	Two-way ANOVA for WT and p11 KO mice			
	Group effect	Two-way ANOVA	*F*_(1,120)_ = 49.4312	*p* < 0.0001
	Time effect	Two-way ANOVA	*F*_(9,120)_ = 9.4748	*p* < 0.0001
	Group-time interaction	Two-way ANOVA	*F*_(9,120)_ = 4.1100	*p* = 0.0001
*C*: DA levels in the NAc with cocaine infusion into the NAc (WT mice)				
	Basal vs 20 min	Mixed linear models	*t*_(54)_ = 1.2	*p* = 0.2351
	Basal vs 40 min	Mixed linear models	*t*_(54)_ = 5.5	*p* < 0.0001
	Basal vs 60 min	Mixed linear models	*t*_(54)_ = 6.54	*p* < 0.0001
	Basal vs 80 min	Mixed linear models	*t*_(54)_ = 7.04	*p* < 0.0001
	Basal vs 100 min	Mixed linear models	*t*_(54)_ = 6.53	*p* < 0.0001
	Basal vs 120 min	Mixed linear models	*t*_(54)_ = 5.23	*p* < 0.0001
	Basal vs 140 min	Mixed linear models	*t*_(54)_ = 4.39	*p* < 0.0001
*C*: DA levels in the NAc with cocaine infusion into the NAc (p11 KO mice)				
	Basal vs 20 min	Mixed linear models	*t*_(54)_ = -0.69	*p* = 0.4942
	Basal vs 40 min	Mixed linear models	*t*_(54)_ = 0.3	*p* = 0.7639
	Basal vs 60 min	Mixed linear models	*t*_(54)_ = 1.54	*p* = 0.1286
	Basal vs 80 min	Mixed linear models	*t*_(54)_ = 1.79	*p* = 0.0783
	Basal vs 100 min	Mixed linear models	*t*_(54)_ = 2.19	*p* = 0.0326
	Basal vs 120 min	Mixed linear models	*t*_(54)_ = 3.78	*p* = 0.0004
	Basal vs 140 min	Mixed linear models	*t*_(54)_ = 2.86	*p* = 0.0059
*D*: DA levels in the NAc with exposure to palatable food				
	Two-way ANOVA for WT and p11 KO mice			
	Group effect	Two-way ANOVA	*F*_(1,120)_ = 37.1184	*p* < 0.0001
	Time effect	Two-way ANOVA	*F*_(9,120)_ = 3.4984	*p* = 0.0007
	Group-time interaction	Two-way ANOVA	*F*_(9,120)_ = 2.3706	*p* = 0.0167
*D*: DA levels in the NAc with exposure to palatable food (WT mice)				
	Basal vs 20 min	Mixed linear models	*t*_(54)_ = 2.08	*p* = 0.0421
	Basal vs 40 min	Mixed linear models	*t*_(54)_ = 4.42	*p* < 0.0001
	Basal vs 60 min	Mixed linear models	*t*_(54)_ = 5.64	*p* < 0.0001
	Basal vs 80 min	Mixed linear models	*t*_(54)_ = 4.7	*p* < 0.0001
	Basal vs 100 min	Mixed linear models	*t*_(54)_ = 3.15	*p* < 0.0001
	Basal vs 120 min	Mixed linear models	*t*_(54)_ = 2.71	*p* < 0.0001
	Basal vs 140 min	Mixed linear models	*t*_(54)_ = 2.23	*p* < 0.0001
*D*: DA levels in the NAc with exposure to palatable food (p11 KO mice)				
	Basal vs 20 min	Mixed linear models	*t*_(54)_ = 1.15	*p* = 0.2544
	Basal vs 40 min	Mixed linear models	*t*_(54)_ = 2.03	*p* = 0.0475
	Basal vs 60 min	Mixed linear models	*t*_(54)_ = 1.72	*p* = 0.0911
	Basal vs 80 min	Mixed linear models	*t*_(54)_ = -0.07	*p* = 0.9414
	Basal vs 100 min	Mixed linear models	*t*_(54)_ = -1.7	*p* = 0.095
	Basal vs 120 min	Mixed linear models	*t*_(54)_ = -0.72	*p* = 0.4769
	Basal vs 140 min	Mixed linear models	*t*_(54)_ = -0.74	*p* = 0.464
*E*: DA levels in the NAc with exposure to female mice				
	Two-way ANOVA for WT and p11 KO mice			
	Group effect	Two-way ANOVA	*F*_(1,80)_ = 39.2674	*p* < 0.0001
	Time effect	Two-way ANOVA	*F*_(7,80)_ = 7.0594	*p* < 0.0001
	Group-time interaction	Two-way ANOVA	*F*_(7,80)_ = 3.7936	*p* = 0.0013
*E*: DA levels in the NAc with exposure to female mice (WT mice)				
	Basal vs 20 min	Mixed linear models	*t*_(35)_ = 5.69	*p* < 0.0001
	Basal vs 40 min	Mixed linear models	*t*_(35)_ = 8.36	*p* < 0.0001
	Basal vs 60 min	Mixed linear models	*t*_(35)_ = 5.29	*p* < 0.0001
	Basal vs 80 min	Mixed linear models	*t*_(35)_ = 3.39	*p* = 0.0017
	Basal vs 100 min	Mixed linear models	*t*_(35)_ = 3.06	*p* = 0.0042

*E*: DA levels in the NAc with exposure to female mice (p11 KO mice)				
	Basal vs 20 min	Mixed linear models	*t*_(35)_ = 0.33	*p* = 0.7429
	Basal vs 40 min	Mixed linear models	*t*_(35)_ = 2.21	*p* = 0.0335
	Basal vs 60 min	Mixed linear models	*t*_(35)_ = 0.46	*p* = 0.6483
	Basal vs 80 min	Mixed linear models	*t*_(35)_ = 0.17	*p* = 0.865
	Basal vs 100 min	Mixed linear models	*t*_(35)_ = -1.9	*p* = 0.5526
*F*: DA levels in the PFC with cocaine infusion				
	Two-way ANOVA for WT and p11 KO mice			
	Group effect	Two-way ANOVA	*F*_(1,120)_ = 0.00970	*p* = 0.7560
	Time effect	Two-way ANOVA	*F*_(9,120)_ = 8.8283	*p* < 0.0001
	Group-time interaction	Two-way ANOVA	*F*_(9,120)_ = 0.00895	*p* = 0.9997
*F*: DA levels in the PFC with cocaine infusion into the PFC (WT mice)				
	Basal vs 20 min	Mixed linear models	*t*_(54)_ = 0.9	*p* = 0.3719
	Basal vs 40 min	Mixed linear models	*t*_(54)_ = 3.04	*p* = 0.0037
	Basal vs 60 min	Mixed linear models	*t*_(54)_ = 4.29	*p* < 0.0001
	Basal vs 80 min	Mixed linear models	*t*_(54)_ = 5.3	*p* < 0.0001
	Basal vs 100 min	Mixed linear models	*t*_(54)_ = 5.98	*p* < 0.0001
	Basal vs 120 min	Mixed linear models	*t*_(54)_ = 4.86	*p* < 0.0001
	Basal vs 140 min	Mixed linear models	*t*_(54)_ = 4.23	*p* < 0.0001
*F*: DA levels in the PFC with cocaine infusion into the PFC (p11 KO mice)				
	Basal vs 20 min	Mixed linear models	*t*_(54)_ = 1.06	*p* = 0.2933
	Basal vs 40 min	Mixed linear models	*t*_(54)_ = 3.26	*p* = 0.0019
	Basal vs 60 min	Mixed linear models	*t*_(54)_ = 4.61	*p* < 0.0001
	Basal vs 80 min	Mixed linear models	*t*_(54)_ = 5.4	*p* < 0.0001
	Basal vs 100 min	Mixed linear models	*t*_(54)_ = 4.94	*p* < 0.0001
	Basal vs 120 min	Mixed linear models	*t*_(54)_ = 5.18	*p* < 0.0001
	Basal vs 140 min	Mixed linear models	*t*_(54)_ = 5.09	*p* < 0.0001
*G*: DA levels in the PFC with exposure to palatable food				
	Two-way ANOVA for WT and p11 KO mice			
	Group effect	Two-way ANOVA	*F*_(1,120)_ = 0.0733	*p* = 0.7870
	Time effect	Two-way ANOVA	*F*_(9,120)_ = 11.4806	*p* < 0.0001
	Group-time interaction	Two-way ANOVA	*F*_(9,120)_ = 0.1100	*p* = 0.9994
*G*: DA levels in the PFC with exposure to palatable food (WT mice)				
	Basal vs 20 min	Mixed linear models	*t*_(54)_ = 4.23	*p* < 0.0001
	Basal vs 40 min	Mixed linear models	*t*_(54)_ = 5.92	*p* < 0.0001
	Basal vs 60 min	Mixed linear models	*t*_(54)_ = 4.67	*p* < 0.0001
	Basal vs 80 min	Mixed linear models	*t*_(54)_ = 4.22	*p* < 0.0001
	Basal vs 100 min	Mixed linear models	*t*_(54)_ = 3.99	*p* = 0.0002
	Basal vs 120 min	Mixed linear models	*t*_(54)_ = 2.54	*p* = 0.0139
	Basal vs 140 min	Mixed linear models	*t*_(54)_ = 1.9	*p* = 0.0631
*G*: DA levels in the PFC with exposure to palatable food (p11 KO mice)				
	Basal vs 20 min	Mixed linear models	*t*_(54)_ = 4.55	*p* < 0.0001
	Basal vs 40 min	Mixed linear models	*t*_(54)_ = 5.86	*p* < 0.0001
	Basal vs 60 min	Mixed linear models	*t*_(54)_ = 5.41	*p* < 0.0001
	Basal vs 80 min	Mixed linear models	*t*_(54)_ = 4.84	*p* < 0.0001
	Basal vs 100 min	Mixed linear models	*t*_(54)_ = 4.42	*p* < 0.0001
	Basal vs 120 min	Mixed linear models	*t*_(54)_ = 3.37	*p* = 0.0014
	Basal vs 140 min	Mixed linear models	*t*_(54)_ = 3.14	*p* = 0.0028
*H*: DA levels in the PFC with exposure to female mice				
	Two-way ANOVA for WT and p11 KO mice			
	Group effect	Two-way ANOVA	*F*_(1,80)_ = 0.1875	*p* = 0.6661
	Time effect	Two-way ANOVA	*F*_(7,80)_ = 12.2601	*p* < 0.0001
	Group-time interaction	Two-way ANOVA	*F*_(7,80)_ = 0.7459	*p* = 0.6339

*H*: DA levels in the PFC with exposure to female mice (WT mice)				
	Basal vs 20 min	Mixed linear models	*t*_(35)_ = 5.76	*p* < 0.0001
	Basal vs 40 min	Mixed linear models	*t*_(35)_ = 7.14	*p* < 0.0001
	Basal vs 60 min	Mixed linear models	*t*_(35)_ = 3.72	*p* = 0.0007
	Basal vs 80 min	Mixed linear models	*t*_(35)_ = 1.28	*p* = 0.2092
	Basal vs 100 min	Mixed linear models	*t*_(35)_ = -0.05	*p* = 0.9614
*H*: DA levels in the PFC with exposure to female mice (p11 KO mice)				
	Basal vs 20 min	Mixed linear models	*t*_(35)_ = 3.34	*p* = 0.002
	Basal vs 40 min	Mixed linear models	*t*_(35)_ = 6.59	*p* < 0.0001
	Basal vs 60 min	Mixed linear models	*t*_(35)_ = 3.54	*p* = 0.0011
	Basal vs 80 min	Mixed linear models	*t*_(35)_ = 1.89	*p* = 0.0675
	Basal vs 100 min	Mixed linear models	*t*_(35)_ = 2.16	*p* = 0.0373
				
Figure 2				
*A*: DA levels in the NAc with cocaine and/or nicotine infusion in p11 KO mice				
	Two-way ANOVA for cocaine and cocaine + nicotine infusion			
	Group effect	Two-way ANOVA	*F*_(1,120)_ = 45.9468	*p* < 0.0001
	Time effect	Two-way ANOVA	*F*_(9,120)_ = 9.0389	*p* < 0.0001
	Group-time interaction	Two-way ANOVA	*F*_(9,120)_ = 3.0465	*p* = 0.0026
	Two-way ANOVA for nicotine and cocaine + nicotine infusion			
	Group effect	Two-way ANOVA	*F*_(1,110)_ = 83.0855	*p* < 0.0001
	Time effect	Two-way ANOVA	*F*_(9,110)_ = 4.9164	*p* < 0.0001
	Group-time interaction	Two-way ANOVA	*F*_(9,110)_ = 5.2703	*p* < 0.0001
*A*: DA levels in the NAc with cocaine and nicotine infusion in p11 KO mice				
	Basal vs 20 min	Mixed linear models	*t*_(54)_ = 1.32	*p* = 0.1932
	Basal vs 40 min	Mixed linear models	*t*_(54)_ = 4.37	*p* < 0.0001
	Basal vs 60 min	Mixed linear models	*t*_(54)_ = 6.19	*p* < 0.0001
	Basal vs 80 min	Mixed linear models	*t*_(54)_ = 6.05	*p* < 0.0001
	Basal vs 100 min	Mixed linear models	*t*_(54)_ = 5.03	*p* < 0.0001
	Basal vs 120 min	Mixed linear models	*t*_(54)_ = 5.53	*p* < 0.0001
	Basal vs 140 min	Mixed linear models	*t*_(54)_ = 5.46	*p* < 0.0001
*A*: DA levels in the NAc with nicotine infusion in p11 KO mice				
	Basal vs 20 min	Mixed linear models	*t*_(45)_ = -0.46	*p* = 0.6509
	Basal vs 40 min	Mixed linear models	*t*_(45)_ = 0.09	*p* = 0.9282
	Basal vs 60 min	Mixed linear models	*t*_(45)_ = 0.21	*p* = 0.8359
	Basal vs 80 min	Mixed linear models	*t*_(45)_ = -0.03	*p* = 0.9802
	Basal vs 100 min	Mixed linear models	*t*_(45)_ = -0.07	*p* = 0.9407
	Basal vs 120 min	Mixed linear models	*t*_(45)_ = 0.08	*p* = 0.9383
	Basal vs 140 min	Mixed linear models	*t*_(45)_ = -1.49	*p* = 0.1437
*B*: DA levels in the NAc with cocaine and/or oxotremorine infusion in p11 KO mice				
	Two-way ANOVA for cocaine and cocaine + oxotremorine infusion			
	Group effect	Two-way ANOVA	*F*_(1,120)_ = 89.7480	*p* < 0.0001
	Time effect	Two-way ANOVA	*F*_(9,120)_ = 13.8003	*p* < 0.0001
	Group-time interaction	Two-way ANOVA	*F*_(9,120)_ = 5.6135	*p* < 0.0001
	Two-way ANOVA for oxotremorine and cocaine + oxotremorine infusion			
	Group effect	Two-way ANOVA	*F*_(1,110)_ = 72.5608	*p* < 0.0001
	Time effect	Two-way ANOVA	*F*_(9,110)_ = 8.88318	*p* < 0.0001
	Group-time interaction	Two-way ANOVA	*F*_(9,110)_ = 5.3849	*p* < 0.0001
*B*: DA levels in the NAc with cocaine and oxotremorine infusion in p11 KO mice				
	Basal vs 20 min	Mixed linear models	*t*_(54)_ = 1.72	*p* = 0.0907
	Basal vs 40 min	Mixed linear models	*t*_(54)_ = 6.21	*p* < 0.0001

	Basal vs 60 min	Mixed linear models	*t*_(54)_ = 7.6	*p* < 0.0001
	Basal vs 80 min	Mixed linear models	*t*_(54)_ = 8.13	*p* < 0.0001
	Basal vs 100 min	Mixed linear models	*t*_(54)_ = 9.28	*p* < 0.0001
	Basal vs 120 min	Mixed linear models	*t*_(54)_ = 7.82	*p* < 0.0001
	Basal vs 140 min	Mixed linear models	*t*_(54)_ = 7.47	*p* < 0.0001
*B*: DA levels in the NAc with oxotremorine infusion in p11 KO mice				
	Basal vs 20 min	Mixed linear models	*t*_(45)_ = 1.25	*p* = 0.2171
	Basal vs 40 min	Mixed linear models	*t*_(45)_ = 1.42	*p* = 0.1612
	Basal vs 60 min	Mixed linear models	*t*_(45)_ = 2.26	*p* = 0.029
	Basal vs 80 min	Mixed linear models	*t*_(45)_ = 1.37	*p* = 0.1774
	Basal vs 100 min	Mixed linear models	*t*_(45)_ = 0.75	*p* = 0.4567
	Basal vs 120 min	Mixed linear models	*t*_(45)_ = 1.88	*p* = 0.0661
	Basal vs 140 min	Mixed linear models	*t*_(45)_ = 0.66	*p* = 0.5144
Figure 3				
*A*: DA levels in the NAc with cocaine infusion				
	Two-way ANOVA for WT and ChAT-p11 cKO mice			
	Group effect	Two-way ANOVA	*F*_(1,140)_ = 108.3406	*p* < 0.0001
	Time effect	Two-way ANOVA	*F*_(9,140)_ = 21.5972	*p* < 0.0001
	Group-time interaction	Two-way ANOVA	*F*_(9,140)_ = 6.8674	*p* < 0.0001
*A*: DA levels in the NAc with cocaine infusion into the NAc (WT mice)				
	Basal vs 20 min	Mixed linear models	*t*_(63)_ = 2.24	*p* = 0.0288
	Basal vs 40 min	Mixed linear models	*t*_(63)_ = 6.09	*p* < 0.0001
	Basal vs 60 min	Mixed linear models	*t*_(63)_ = 8.34	*p* < 0.0001
	Basal vs 80 min	Mixed linear models	*t*_(63)_ = 9.09	*p* < 0.0001
	Basal vs 100 min	Mixed linear models	*t*_(63)_ = 9	*p* < 0.0001
	Basal vs 120 min	Mixed linear models	*t*_(63)_ = 9.97	*p* < 0.0001
	Basal vs 140 min	Mixed linear models	*t*_(63)_ = 9.71	*p* < 0.0001
*A*: DA levels in the NAc with cocaine infusion into the NAc (ChAT-p11 cKO mice)				
	Basal vs 20 min	Mixed linear models	*t*_(63)_ = 0.28	*p* = 0.7771
	Basal vs 40 min	Mixed linear models	*t*_(63)_ = 4.04	*p* = 0.0001
	Basal vs 60 min	Mixed linear models	*t*_(63)_ = 4.2	*p* < 0.0001
	Basal vs 80 min	Mixed linear models	*t*_(63)_ = 4.44	*p* < 0.0001
	Basal vs 100 min	Mixed linear models	*t*_(63)_ = 4.82	*p* < 0.0001
	Basal vs 120 min	Mixed linear models	*t*_(63)_ = 4.84	*p* < 0.0001
	Basal vs 140 min	Mixed linear models	*t*_(63)_ = 5.38	*p* < 0.0001
*B*: DA levels in the NAc with exposure to palatable food				
	Two-way ANOVA for WT and ChAT-p11 cKO mice			
	Group effect	Two-way ANOVA	*F*_(1,140)_ = 29.2503	*p* < 0.0001
	Time effect	Two-way ANOVA	*F*_(9,140)_ = 2.5700	*p* = 0.0091
	Group-time interaction	Two-way ANOVA	*F*_(9,140)_ = 3.0056	*p* = 0.0026
*B*: DA levels in the NAc with exposure to palatable food (WT mice)				
	Basal vs 20 min	Mixed linear models	*t*_(63)_ = 5.14	*p* < 0.0001
	Basal vs 40 min	Mixed linear models	*t*_(63)_ = 6.24	*p* < 0.0001
	Basal vs 60 min	Mixed linear models	*t*_(63)_ = 2.91	*p* = 0.005
	Basal vs 80 min	Mixed linear models	*t*_(63)_ = 1.95	*p* = 0.0554
	Basal vs 100 min	Mixed linear models	*t*_(63)_ = 1.96	*p* = 0.0541
	Basal vs 120 min	Mixed linear models	*t*_(63)_ = 1.08	*p* = 0.284
	Basal vs 140 min	Mixed linear models	*t*_(63)_ = 1.77	*p* = 0.0816
*B*: DA levels in the NAc with exposure to palatable food (ChAT-p11 cKO mice)				
	Basal vs 20 min	Mixed linear models	*t*_(63)_ = -0.43	*p* = 0.6675
	Basal vs 40 min	Mixed linear models	*t*_(63)_ = -0.31	*p* = 0.7584
	Basal vs 60 min	Mixed linear models	*t*_(63)_ = -0.45	*p* = 0.6577
	Basal vs 80 min	Mixed linear models	*t*_(63)_ = -0.72	*p* = 0.4734

	Basal vs 100 min	Mixed linear models	*t*_(63)_ = 0.13	*p* = 0.9005
	Basal vs 120 min	Mixed linear models	*t*_(63)_ = 0.2	*p* = 0.8398
	Basal vs 140 min	Mixed linear models	*t*_(63)_ = -0.31	*p* = 0.7576
*C*: DA levels in the NAc with exposure to female mice				
	Two-way ANOVA for WT and ChAT-p11 cKO mice			
	Group effect	Two-way ANOVA	*F*_(1,112)_ = 31.1748	*p* < 0.0001
	Time effect	Two-way ANOVA	*F*_(7,112)_ = 6.5263	*p* < 0.0001
	Group-time interaction	Two-way ANOVA	*F*_(7,112)_ = 4.9259	*p* < 0.0001
*C*: DA levels in the NAc with exposure to female mice (WT mice)				
	Basal vs 20 min	Mixed linear models	*t*_(49)_ = 5	*p* < 0.0001
	Basal vs 40 min	Mixed linear models	*t*_(49)_ = 6.39	*p* < 0.0001
	Basal vs 60 min	Mixed linear models	*t*_(49)_ = 3.29	*p* = 0.0018
	Basal vs 80 min	Mixed linear models	*t*_(49)_ = 1.8	*p* = 0.0785
	Basal vs 100 min	Mixed linear models	*t*_(49)_ = 1.79	*p* = 0.0802
*C*: DA levels in the NAc with exposure to female mice (ChAT-p11 cKO mice)				
	Basal vs 20 min	Mixed linear models	*t*_(49)_ = 0.37	*p* = 0.7103
	Basal vs 40 min	Mixed linear models	*t*_(49)_ = 1.12	*p* = 0.2681
	Basal vs 60 min	Mixed linear models	*t*_(49)_ = 2.51	*p* = 0.0153
	Basal vs 80 min	Mixed linear models	*t*_(49)_ = 1.24	*p* = 0.2202
	Basal vs 100 min	Mixed linear models	*t*_(49)_ = 0.02	*p* = 0.9862
Figure 4				
*C*: DA levels in the NAc with cocaine infusion in ChAT-p11 cKO mice injected with *AAV-p11* or *AAV-YFP*				
	Two-way ANOVA for *AAV-p11* and *AAV-YFP*			
	Group effect	Two-way ANOVA	*F*_(1,140)_ = 39.4565	*p* < 0.0001
	Time effect	Two-way ANOVA	*F*_(9,140)_ = 8.6938	*p* < 0.0001
	Group-time interaction	Two-way ANOVA	*F*_(9,140)_ = 2.6737	*p* < 0.0001
*C*: DA levels in the NAc with cocaine infusion into the NAc (p11 cKO + *AAV-YFP*)				
	Basal vs 20 min	Mixed linear models	*t*_(63)_ = 0.17	*p* = 0.8632
	Basal vs 40 min	Mixed linear models	*t*_(63)_ = 4.83	*p* < 0.0001
	Basal vs 60 min	Mixed linear models	*t*_(63)_ = 5.37	*p* < 0.0001
	Basal vs 80 min	Mixed linear models	*t*_(63)_ = 5.71	*p* < 0.0001
	Basal vs 100 min	Mixed linear models	*t*_(63)_ = 6.91	*p* < 0.0001
	Basal vs 120 min	Mixed linear models	*t*_(63)_ = 5.5	*p* < 0.0001
	Basal vs 140 min	Mixed linear models	*t*_(63)_ = 4.29	*p* < 0.0001
*C*: DA levels in the NAc with cocaine infusion into the NAc (p11 cKO + *AAV-p11*)				
	Basal vs 20 min	Mixed linear models	*t*_(63)_ = 0.91	*p* = 0.3647
	Basal vs 40 min	Mixed linear models	*t*_(63)_ = 4.15	*p* = 0.0001
	Basal vs 60 min	Mixed linear models	*t*_(63)_ = 5.59	*p* < 0.0001
	Basal vs 80 min	Mixed linear models	*t*_(63)_ = 6.41	*p* < 0.0001
	Basal vs 100 min	Mixed linear models	*t*_(63)_ = 6.22	*p* < 0.0001
	Basal vs 120 min	Mixed linear models	*t*_(63)_ = 5.92	*p* < 0.0001
	Basal vs 140 min	Mixed linear models	*t*_(63)_ = 4.92	*p* < 0.0001
*D*: DA levels in the NAc with exposure to palatable food in ChAT-p11 cKO mice injected with *AAV-p11* or *AAV-YFP*				
	Two-way ANOVA for *AAV-p11* and *AAV-YFP*			
	Group effect	Two-way ANOVA	*F*_(1,140)_ = 57.9163	*p* < 0.0001
	Time effect	Two-way ANOVA	*F*_(9,140)_ = 3.8107	*p* = 0.0003
	Group-time interaction	Two-way ANOVA	*F*_(9,140)_ = 3.4534	*p* = 0.0007
*D*: DA levels in the NAc with exposure to palatable food (p11 cKO + *AAV-YFP*)				
	Basal vs 20 min	Mixed linear models	*t*_(63)_ = -0.54	*p* = 0.5909
	Basal vs 40 min	Mixed linear models	*t*_(63)_ = 0.75	*p* = 0.4536
	Basal vs 60 min	Mixed linear models	*t*_(63)_ = -2.08	*p* = 0.0412

	Basal vs 80 min	Mixed linear models	*t*_(63)_ = -2.62	*p* = 0.0111
	Basal vs 100 min	Mixed linear models	*t*_(63)_ = -2.21	*p* = 0.0308
	Basal vs 120 min	Mixed linear models	*t*_(63)_ = -1.85	*p* = 0.0695
	Basal vs 140 min	Mixed linear models	*t*_(63)_ = -3.65	*p* = 0.0005
*D*: DA levels in the NAc with exposure to palatable food (p11 cKO + *AAV-p11*)				
	Basal vs 20 min	Mixed linear models	*t*_(63)_ = 4.45	*p* < 0.0001
	Basal vs 40 min	Mixed linear models	*t*_(63)_ = 6.65	*p* < 0.0001
	Basal vs 60 min	Mixed linear models	*t*_(63)_ = 3.76	*p* = 0.0004
	Basal vs 80 min	Mixed linear models	*t*_(63)_ = 3.66	*p* = 0.0005
	Basal vs 100 min	Mixed linear models	*t*_(63)_ = 2.07	*p* = 0.0424
	Basal vs 120 min	Mixed linear models	*t*_(63)_ = 2.2	*p* = 0.0315
	Basal vs 140 min	Mixed linear models	*t*_(63)_ = 0.98	*p* = 0.3308
*E*: DA levels in the NAc with exposure to female mice in ChAT-p11 cKO mice injected with *AAV-p11* or *AAV-YFP*				
	Two-way ANOVA for *AAV-p11* and *AAV-YFP*			
	Group effect	Two-way ANOVA	*F*_(1,112)_ = 25.2729	*p* < 0.0001
	Time effect	Two-way ANOVA	*F*_(7,112)_ = 5.4068	*p* < 0.0001
	Group-time interaction	Two-way ANOVA	*F*_(7,112)_ = 2.5674	*p* = 0.0172
*E*: DA levels in the NAc with exposure to female mice (p11 cKO + *AAV-YFP*)				
	Basal vs 20 min	Mixed linear models	*t*_(49)_ = 0.62	*p* = 0.5353
	Basal vs 40 min	Mixed linear models	*t*_(49)_ = 1.92	*p* = 0.0608
	Basal vs 60 min	Mixed linear models	*t*_(49)_ = 0.25	*p* = 0.8038
	Basal vs 80 min	Mixed linear models	*t*_(49)_ = -1.14	*p* = 0.2615
	Basal vs 100 min	Mixed linear models	*t*_(49)_ = -0.64	*p* = 0.5276
*E*: DA levels in the NAc with exposure to female mice (p11 cKO + *AAV-p11*)				
	Basal vs 20 min	Mixed linear models	*t*_(49)_ = 5.9	*p* < 0.0001
	Basal vs 40 min	Mixed linear models	*t*_(49)_ = 5.69	*p* < 0.0001
	Basal vs 60 min	Mixed linear models	*t*_(49)_ = 3.21	*p* = 0.0023
	Basal vs 80 min	Mixed linear models	*t*_(49)_ = 2.05	*p* = 0.0458
	Basal vs 100 min	Mixed linear models	*t*_(49)_ = 2.38	*p* = 0.0212
Figure 5				
*A*: ACh levels in the NAc with cocaine infusion				
	Two-way ANOVA for WT and ChAT-p11 cKO mice			
	Group effect	Two-way ANOVA	*F*_(1,324 )_ = 35.3923	*p* < 0.0001
	Time effect	Two-way ANOVA	*F*_(17,324 )_ = 0.9289	*p* = 0.5400
	Group-time interaction	Two-way ANOVA	*F*_(17,324)_ = 1.7341	*p* = 0.0358
*A*: ACh levels in the NAc with cocaine infusion into the NAc (WT mice)				
	Basal vs 10 min	Mixed linear models	*t*_(187)_ = -0.14	*p* = 0.8895
	Basal vs 20 min	Mixed linear models	*t*_(187)_ = 4.07	*p* < 0.0001
	Basal vs 30 min	Mixed linear models	*t*_(187)_ = 2.86	*p* = 0.0047
	Basal vs 40 min	Mixed linear models	*t*_(187)_ = 2.65	*p* = 0.0087
	Basal vs 50 min	Mixed linear models	*t*_(187)_ = 3.73	*p* = 0.0003
	Basal vs 60 min	Mixed linear models	*t*_(187)_ = 3.55	*p* = 0.0005
	Basal vs 70 min	Mixed linear models	*t*_(187)_ = 4.11	*p* < 0.0001
	Basal vs 80 min	Mixed linear models	*t*_(187)_ = 3.23	*p* = 0.0015
	Basal vs 90 min	Mixed linear models	*t*_(187)_ = 2.53	*p* = 0.0123
	Basal vs 100 min	Mixed linear models	*t*_(187)_ = 2.92	*p* = 0.0039
	Basal vs 110 min	Mixed linear models	*t*_(187)_ = 2.76	*p* = 0.0063
	Basal vs 120 min	Mixed linear models	*t*_(187)_ = 3.29	*p* = 0.0012
*A*: ACh levels in the NAc with cocaine infusion into the NAc (ChAT-p11 cKO mice)				
	Basal vs 10 min	Mixed linear models	*t*_(119)_ = -0.55	*p* = 0.5808
	Basal vs 20 min	Mixed linear models	*t*_(119)_ = -1.2	*p* = 0.2334
	Basal vs 30 min	Mixed linear models	*t*_(119)_ = -1.98	*p* = 0.0502

	Basal vs 40 min	Mixed linear models	*t*_(119)_ = -2.38	*p* = 0.0191
	Basal vs 50 min	Mixed linear models	*t*_(119)_ = -0.46	*p* = 0.6487
	Basal vs 60 min	Mixed linear models	*t*_(119)_ = 0.21	*p* = 0.835
	Basal vs 70 min	Mixed linear models	*t*_(119)_ = -0.44	*p* = 0.6587
	Basal vs 80 min	Mixed linear models	*t*_(119)_ = -1.03	*p* = 0.3053
	Basal vs 90 min	Mixed linear models	*t*_(119)_ = -1.36	*p* = 0.1767
	Basal vs 100 min	Mixed linear models	*t*_(119)_ = -0.44	*p* = 0.6627
	Basal vs 110 min	Mixed linear models	*t*_(119)_ = 0.07	*p* = 0.9468
	Basal vs 120 min	Mixed linear models	*t*_(119)_ = 0.39	*p* = 0.6987
Figure 6				
*B*: DA levels in the NAc with CNO infusion in ChAT-p11 cKO mice injected with *AAV-rM3D* or *AAV-mCherry*		One-way ANOVA	*F*_(3,20)_ = 7.643	*p* = 0.0014
	*AAV-mCherry*/CNO 10 µM vs *AAV-rM3D*/CNO 10 µM	Newman–Keuls *post hoc* test		*p* < 0.01
	*AAV-rM3D*/CNO 3 µM vs *AAV-rM3D*/CNO 10 µM	Newman–Keuls *post hoc* test		*p* < 0.01
*C*: DA levels in the NAc with cocaine or CNO + cocaine infusion in ChAT-p11 cKO mice injected with *AAV-rM3D* or *AAV-mCherry*				
	Two-way ANOVA for *AAV-rM3D*/CNO + cocaine or *AAV-mCherry*/CNO + cocaine			
	Group effect	Two-way ANOVA	*F*_(1,100)_ = 94.7020	*p* < 0.0001
	Time effect	Two-way ANOVA	*F*_(9,100)_ = 23.4516	*p* < 0.0001
	Group-time interaction	Two-way ANOVA	*F*_(9,100)_ = 5.7876	*p* < 0.0001
	Two-way ANOVA for *AAV-rM3D*/CNO + cocaine or *AAV-rM3D*/cocaine			
	Group effect	Two-way ANOVA	*F*_(1,100)_ = 106.4829	*p* < 0.0001
	Time effect	Two-way ANOVA	*F*_(9,100)_ = 15.2109	*p* < 0.0001
	Group-time interaction	Two-way ANOVA	*F*_(9,100)_ = 6.4710	*p* < 0.0001
*C*: DA levels in the NAc with CNO + cocaine infusion in ChAT-p11 cKO mice injected with *AAV-rM3D*				
	Basal vs 20 min	Mixed linear models	*t*_(45)_ = 2.53	*p* = 0.015
	Basal vs 40 min	Mixed linear models	*t*_(45)_ = 7.21	*p* < 0.0001
	Basal vs 60 min	Mixed linear models	*t*_(45)_ = 6.78	*p* < 0.0001
	Basal vs 80 min	Mixed linear models	*t*_(45)_ = 7.95	*p* < 0.0001
	Basal vs 100 min	Mixed linear models	*t*_(45)_ = 8.68	*p* < 0.0001
	Basal vs 120 min	Mixed linear models	*t*_(45)_ = 7.87	*p* < 0.0001
	Basal vs 140 min	Mixed linear models	*t*_(45)_ = 10.11	*p* < 0.0001
*C*: DA levels in the NAc with CNO + cocaine infusion in ChAT-p11 cKO mice injected with *AAV-mCherry*				
	Basal vs 20 min	Mixed linear models	*t*_(45)_ = 0.64	*p* = 0.5243
	Basal vs 40 min	Mixed linear models	*t*_(45)_ = 3.98	*p* = 0.0002
	Basal vs 60 min	Mixed linear models	*t*_(45)_ = 6.48	*p* < 0.0001
	Basal vs 80 min	Mixed linear models	*t*_(45)_ = 5.56	*p* < 0.0001
	Basal vs 100 min	Mixed linear models	*t*_(45)_ = 6.59	*p* < 0.0001
	Basal vs 120 min	Mixed linear models	*t*_(45)_ = 6.36	*p* < 0.0001
	Basal vs 140 min	Mixed linear models	*t*_(45)_ = 6.33	*p* < 0.0001
*C*: DA levels in the NAc with cocaine infusion in ChAT-p11 cKO mice injected with *AAV-rM3D*				
	Basal vs 20 min	Mixed linear models	*t*_(45)_ = -0.45	*p* = 0.6554
	Basal vs 40 min	Mixed linear models	*t*_(45)_ = 2.43	*p* = 0.0192
	Basal vs 60 min	Mixed linear models	*t*_(45)_ = 2.96	*p* = 0.0049
	Basal vs 80 min	Mixed linear models	*t*_(45)_ = 1.85	*p* = 0.0709
	Basal vs 100 min	Mixed linear models	*t*_(45)_ = 1.59	*p* = 0.1184
	Basal vs 120 min	Mixed linear models	*t*_(45)_ = 2.56	*p* = 0.014
	Basal vs 140 min	Mixed linear models	*t*_(45)_ = 2.99	*p* = 0.0045
*C*: DA levels in the NAc with cocaine infusion in ChAT-p11 cKO mice injected with *AAV-mCherry*				
	Basal vs 20 min	Mixed linear models	*t*_(45)_ = 0.81	*p* = 0.4199
	Basal vs 40 min	Mixed linear models	*t*_(45)_ = 0.81	*p* = 0.0002

	Basal vs 60 min	Mixed linear models	*t*_(45)_ = 0.81	*p* < 0.0001
	Basal vs 80 min	Mixed linear models	*t*_(45)_ = 0.81	*p* < 0.0001
	Basal vs 100 min	Mixed linear models	*t*_(45)_ = 0.81	*p* < 0.0001
	Basal vs 120 min	Mixed linear models	*t*_(45)_ = 0.81	*p* < 0.0001
	Basal vs 140 min	Mixed linear models	*t*_(45)_ = 0.81	*p* < 0.0001
Figure 7				
DA levels in the NAc with cocaine or CNO + cocaine infusion in ChAT-p11 cKO mice injected with *AAV-hM4D* or *AAV-mCherry*				
	Two-way ANOVA for *AAV-hM4D*/CNO + cocaine or *AAV-mCherry*/CNO + cocaine			
	Group effect	Two-way ANOVA	*F*_(1,150)_ = 12.3097	*p* = 0.0006
	Time effect	Two-way ANOVA	*F*_(9,150)_ = 17.6639	*p* < 0.0001
	Group-time interaction	Two-way ANOVA	*F*_(9,150)_ = 1.2133	*p* = 0.2908
	Two-way ANOVA for *AAV-hM4D*/CNO + cocaine or *AAV-hM4D*/cocaine			
	Group effect	Two-way ANOVA	*F*_(1,220)_ = 32.4559	*p* < 0.0001
	Time effect	Two-way ANOVA	*F*_(9,220)_ = 14.3297	*p* < 0.0001
	Group-time interaction	Two-way ANOVA	*F*_(9,220)_ = 1.7342	*p* = 0.0826
DA levels in the NAc with CNO + cocaine infusion in ChAT-p11 cKO mice injected with *AAV-hM4D*				
	Basal vs 20 min	Mixed linear models	*t*_(90)_ = 2.68	*p* = 0.0087
	Basal vs 40 min	Mixed linear models	*t*_(90)_ = 4.63	*p* < 0.0001
	Basal vs 60 min	Mixed linear models	*t*_(90)_ = 7.40	*p* < 0.0001
	Basal vs 80 min	Mixed linear models	*t*_(90)_ = 7.17	*p* < 0.0001
	Basal vs 100 min	Mixed linear models	*t*_(90)_ = 6.77	*p* < 0.0001
	Basal vs 120 min	Mixed linear models	*t*_(90)_ = 6.99	*p* < 0.0001
	Basal vs 140 min	Mixed linear models	*t*_(90)_ = 7.22	*p* < 0.0001
DA levels in the NAc with CNO + cocaine infusion in ChAT-p11 cKO mice injected with *AAV-mCherry*				
	Basal vs 20 min	Mixed linear models	*t*_(45)_ = -0.11	*p* = 0.9112
	Basal vs 40 min	Mixed linear models	*t*_(45)_ = 3.79	*p* = 0.0005
	Basal vs 60 min	Mixed linear models	*t*_(45)_ = 6.43	*p* < 0.0001
	Basal vs 80 min	Mixed linear models	*t*_(45)_ = 5.87	*p* < 0.0001
	Basal vs 100 min	Mixed linear models	*t*_(45)_ = 5.20	*p* < 0.0001
	Basal vs 120 min	Mixed linear models	*t*_(45)_ = 5.13	*p* < 0.0001
	Basal vs 140 min	Mixed linear models	*t*_(45)_ = 5.28	*p* < 0.0001
DA levels in the NAc with cocaine infusion in ChAT-p11 cKO mice injected with *AAV-hM4D*				
	Basal vs 20 min	Mixed linear models	*t*_(108)_ = 4.40	*p* < 0.0001
	Basal vs 40 min	Mixed linear models	*t*_(108)_ = 7.77	*p* < 0.0001
	Basal vs 60 min	Mixed linear models	*t*_(108)_ = 8.47	*p* < 0.0001
	Basal vs 80 min	Mixed linear models	*t*_(108)_ = 8.23	*p* < 0.0001
	Basal vs 100 min	Mixed linear models	*t*_(108)_ = 8.63	*p* < 0.0001
	Basal vs 120 min	Mixed linear models	*t*_(108)_ = 8.33	*p* < 0.0001
	Basal vs 140 min	Mixed linear models	*t*_(108)_ = 7.47	*p* < 0.0001
DA levels in the NAc with cocaine infusion in ChAT-p11 cKO mice injected with *AAV-mCherry*				
	Basal vs 20 min	Mixed linear models	*t*_(63)_ = 0.81	*p* = 0.4199
	Basal vs 40 min	Mixed linear models	*t*_(63)_ = 4.02	*p* = 0.0002
	Basal vs 60 min	Mixed linear models	*t*_(63)_ = 5.64	*p* < 0.0001
	Basal vs 80 min	Mixed linear models	*t*_(63)_ = 6.52	*p* < 0.0001
	Basal vs 100 min	Mixed linear models	*t*_(63)_ = 5.89	*p* < 0.0001
	Basal vs 120 min	Mixed linear models	*t*_(63)_ = 5.89	*p* < 0.0001
	Basal vs 140 min	Mixed linear models	*t*_(63)_ = 6.00	*p* < 0.0001
Table 1				
Basal levels of dopamine, dopamine metabolites, and ACh				
	NAc DA, WT vs p11 KO	*t* test	*t*_(39)_ = 0.1283	*p* = 0.8986
	NAc DOPAC, WT vs p11 KO	*t* test	*t*_(35)_ = 0.04948	*p* = 0.9608
	NAc HVA, WT vs p11 KO	*t* test	*t*_(22)_ = 0.2247	*p* = 0.8243

	PFC DA, WT vs p11 KO	*t* test (Welch's-correction)	*t*_(13)_ = 1.183	*p* = 0.2672
	PFC DOPAC, WT vs p11 KO	*t* test	*t*_(12)_ = 0.6739	*p* = 0.5132
	PFC HVA, WT vs p11 KO	*t* test	*t*_(5)_ = 0.1866	*p* = 0.8593
	NAc DA, WT vs ChAT-p11 cKO	*t* test	*t*_(14)_ = 1.139	*p* = 0.2739
	NAc DOPAC, WT vs ChAT-p11 cKO	*t* test	*t*_(14)_ = 0.04194	*p* = 0.9671
	NAc ACh, WT vs ChAT-p11 cKO	*t* test	*t*_(18)_ = 0.9686	*p* = 0.3456
	NAc DA, ChAT-p11 cKO + *AAV-YFP* vs ChAT-p11 cKO + *AAV-p11*	*t* test	*t*_(14)_ = 0.5145	*p* = 0.6149
	NAc DA, ChAT-p11 cKO + *AAV-mCherry* vs ChAT-p11 cKO + *AAV-rM3D(Gs)*	*t* test	*t*_(10)_ = 0.1095	*p* = 0.9150
	NAc DA, ChAT-p11 cKO + *AAV-mCherry* vs ChAT-p11 cKO + *AAV-hM4D (Gi)*	*t* test	*t*_(19)_ = 1.011	*p* = 0.3246

## Results

### Dopamine responses to rewarding stimuli in the NAc and PFC of constitutive p11 KO mice

The levels of dopamine in the NAc in response to a drug of abuse, cocaine, and exposure to natural rewarding stimuli, a palatable food or female mouse, were determined with *in vivo* microdialysis. The basal extracellular levels of dopamine and its metabolites [3,4-dihydroxy-phenylacetic acid (DOPAC) and homovanillic acid (HVA)] in the NAc and PFC were similar between wild-type (WT) and constitutive p11 KO (p11 KO) mice (Table 1). Cocaine infusion (1 µM) into the NAc increased the levels of dopamine to 150% of control in the NAc of WT mice, but the dopamine response to cocaine infusion was largely attenuated in p11 KO mice (Fig. 1*C*). Exposure to a palatable food or female mouse increased the dopamine levels similarly to cocaine infusion in the NAc of WT mice (Fig. 1*D*,*E*). The dopamine response to the palatable food or female mouse was abolished in the NAc of p11 KO mice. In the PFC, all the rewarding stimuli increased the dopamine levels to the same extent in WT and p11 KO mice (Fig. 1*F–H*). These results indicate that p11 is selectively involved in the regulation of the mesolimbic (VTA-NAc) dopamine system, but not in the regulation of the mesocortical (VTA-PFC) dopamine system.

### Effects of a nicotinic or muscarinic receptor agonist on the attenuated dopamine response to cocaine in the NAc of constitutive p11 KO mice

**Figure 2.**
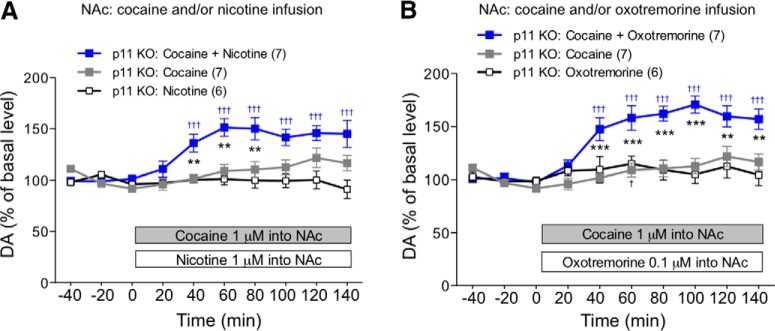
The dopamine (DA) response to cocaine infusion in the NAc in constitutive p11 KO mice is restored by nicotinic or muscarinic receptor stimulation in the NAc. Effects of local infusion of cocaine (1 µM) and/or nicotine (1 µM) (***A***) or cocaine (1 µM) and/or non-selective muscarinic receptor agonist, oxotremorine (0.1 µM) (***B***) into the NAc on the extracellular levels of DA in the NAc of constitutive p11 KO mice. The dose of nicotine or oxotremorine without effects on the dopamine levels was used. Data for cocaine infusion alone were reproduced from Figure 1*C* for comparison. The basal values for each group were obtained as the average of three stable baseline samples, and all values are calculated as a percentage of the basal values within the same group (100%). Data represent mean ± SEM. **p < 0.01, ***p < 0.001 versus the cocaine group; two-way ANOVA and Bonferroni multiple comparison test. ^†^*p* < 0.05, ^†††^*p* < 0.001 versus the basal levels of DA in the same group. The number of mice is indicated in parentheses under each experimental condition.

p11 is highly expressed in NAc CINs (Warner-Schmidt et al., 2012) and is involved in the regulation of ACh release (Virk et al., 2016). In addition, ACh has been shown to stimulate dopamine release via activation of α4β2 nicotinic ACh receptors (nAChRs) (Wonnacott et al., 2000; Hamada et al., 2004) and M5 muscarinic receptors (mAChRs) (Bendor et al., 2010; Kuroiwa et al., 2012) at dopaminergic axon terminals. These observations suggest that p11 regulates mesolimbic dopamine release by regulating cholinergic signaling at dopaminergic axon terminals. We therefore investigated whether activation of nAChRs or mAChRs could restore the dopamine responses to cocaine in the NAc of p11 KO mice (Fig. 2*A*,*B*). When cocaine was co-infused into the NAc (1 µM) with either nicotine (1 µM) or the non-selective mAChR agonist, oxotremorine (0.1 µM), it was able to increase the dopamine levels in the NAc of p11 KO mice, similarly to those of WT mice. Infusion of either nicotine (1 µM) or oxotremorine (0.1 µM) alone did not affect the levels of dopamine in the NAc of WT or p11 KO mice. These results suggest that lack of p11 may reduce ACh release and ACh-mediated effects, resulting in the attenuation of the dopamine responses to cocaine in the NAc of p11 KO mice.

### Role of p11 in NAc CINs in the dopamine responses to rewarding stimuli

**Figure 3.**
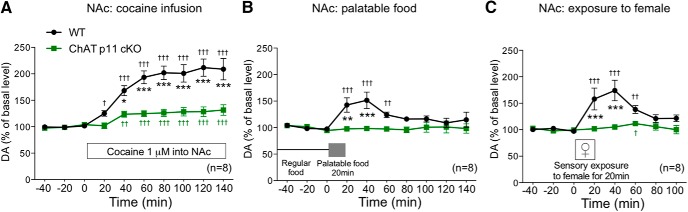
The dopamine (DA) response to rewarding stimuli in the NAc of ChAT-p11 conditional KO (cKO) mice. The effects of cocaine infusion (1 µM) into the NAc (***A***), exposure to palatable food (***B***), and exposure to female mice (***C***) on the extracellular levels of DA in the NAc of WT (ChAT-Cre^-/-^ p11^flox/flox^) and ChAT-p11 cKO (ChAT-Cre^+^ p11^flox/flox^) mice. The basal values for each group were obtained as the average of three stable baseline samples, and all values are calculated as a percentage of the basal values within the same group (100%). Data represent mean ± SEM. *p < 0.05, **p < 0.01, ***p < 0.001 versus WT mice; two-way ANOVA and Bonferroni multiple comparison test. ^†^*p* < 0.05, ^††^*p* < 0.01, ^†††^*p* < 0.001 versus the basal levels of DA in the same group. The number of mice is indicated in parentheses.

To directly investigate the role of p11 in ChAT-expressing cells, the dopamine responses to rewarding stimuli were evaluated in the NAc of ChAT cell-specific p11 KO mice (ChAT-p11 cKO mice), which were obtained by mating p11 floxed mice with ChAT-Cre mice (Warner-Schmidt et al., 2012). The basal extracellular levels of dopamine in the NAc were not affected by deletion of p11 in ChAT cells (Table 1). Cocaine infusion (1 µM) into the NAc or exposure to a palatable food or female mouse increased the extracellular levels of dopamine in the NAc of control mice (ChAT-Cre^-/-^ p11^flox/flox^ mice; Fig. 3). The dopamine responses to the rewarding stimuli were attenuated or completely abolished in the NAc of ChAT-p11 cKO mice (ChAT-Cre^+^ p11^flox/flox^ mice). These results indicate that p11 in ChAT cells plays a critical role in the dopamine responses to rewarding stimuli.

**Figure 4.**
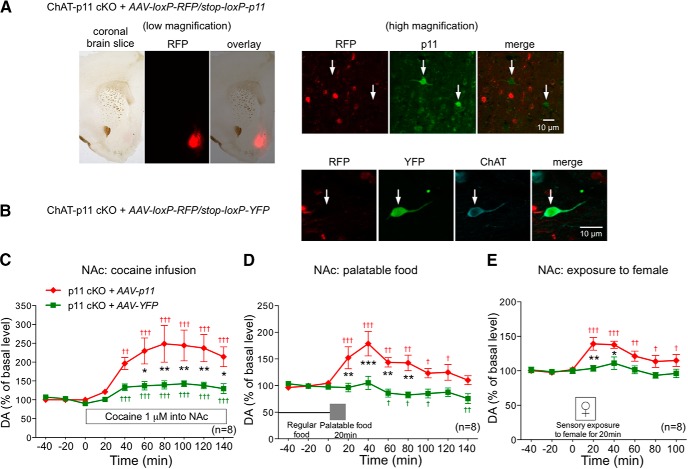
Overexpression of p11 in ChAT cells of the NAc restores the dopamine (DA) response to rewarding stimuli in ChAT p11 cKO mice. ***A***, Immunohistochemical detection of RFP (red) and p11 (green) in the NAc of ChAT-p11 cKO mice injected with p11-overexpressing virus [*AAV-loxP-RFP/stop-loxP-p11* (*AAV-p11*)] into the NAc. RFP is expressed in ChAT-Cre^-/-^ cells, and p11 was expressed in ChAT-Cre^+^ cells. In images with low magnification (left panel), RFP-positive area in the shell of the NAc corresponds to the area of viral injection. In images with high magnification (right panel), p11 is overexpressed in RFP-negative neurons. Arrows indicate cells overexpressing p11. ***B***, Immunohistochemical detection of RFP (red), YFP (green), and ChAT (blue) in the NAc of ChAT-p11 cKO mice injected with control virus [*AAV-loxP-RFP/stop-loxP-YFP* (*AAV-YFP*)]. RFP was expressed in ChAT-Cre^-/-^ cells, and YFP was expressed in ChAT-Cre^+^ cells. YFP expression overlapped with ChAT staining. Arrow indicates ChAT-positive CINs expressing YFP. ***C–E***, The effects of cocaine infusion (1 µM) into the NAc (***C***), exposure to palatable food (***D***), and exposure to female mice (***E***) on the extracellular levels of DA in the NAc of ChAT-p11 cKO mice injected with control (AAV-YFP) or p11-overexpressing (AAV-p11) virus. The basal values for each group were obtained as the average of three stable baseline samples, and all values are calculated as a percentage of the basal values within the same group (100%). Data represent mean ± SEM. *p < 0.05, **p < 0.01, ***p < 0.001 versus ChAT-p11 cKO mice with control virus injection; two-way ANOVA and Bonferroni multiple comparison test. ^†^*p* < 0.05, ^††^*p* < 0.01, ^†††^*p* < 0.001 versus the basal levels of DA in the same group. The number of mice is indicated in parentheses.

ChAT-positive cells or axon fibers in the NAc correspond to CINs, and therefore p11 in NAc CINs likely regulates the dopamine responses. However, there is a possibility that p11 expressed in ChAT cells of other brain regions such as basal forebrain cholinergic neurons and pontomesencephalic cholinergic neurons may indirectly affect the VTA-NAc dopamine system. To rule out this possibility, p11 was overexpressed in CINs by injecting *AAV-loxP-RFP/stop-loxP-p11* (*AAV-p11*) in the NAc of ChAT-p11 cKO mice (Warner-Schmidt et al., 2012), and the dopamine responses to rewarding stimuli were evaluated. Injection of p11-overexpressing virus (*AAV-p11*) into the NAc induced the expression of RFP in ChAT-Cre^-/-^ cells such as medium-sized spiny neurons and GABAergic interneurons (Fig. 4*A*). In ChAT-Cre^+^ cells, p11 was expressed in RFP-negative large-sized neurons. As control virus, *AAV-loxP-RFP/stop-loxP-YFP* (*AAV-YFP*) was injected into the NAc. YFP expression induced by ChAT-Cre was indeed observed in RFP-negative ChAT expressing cells (Fig. 4*B*). These immunohistochemical analyses revealed that p11 is selectively overexpressed in NAc CINs. Overexpression of p11, but not of YFP, in NAc CINs restored the dopamine responses to rewarding stimuli in the NAc of ChAT-p11 cKO mice (Fig. 4*C–E*). These results suggest that NAc CINs have the ability to regulate the mesolimbic dopamine reward system via p11-dependent mechanisms.

### Role of p11 in NAc CINs in the cocaine-induced ACh release

**Figure 5.**
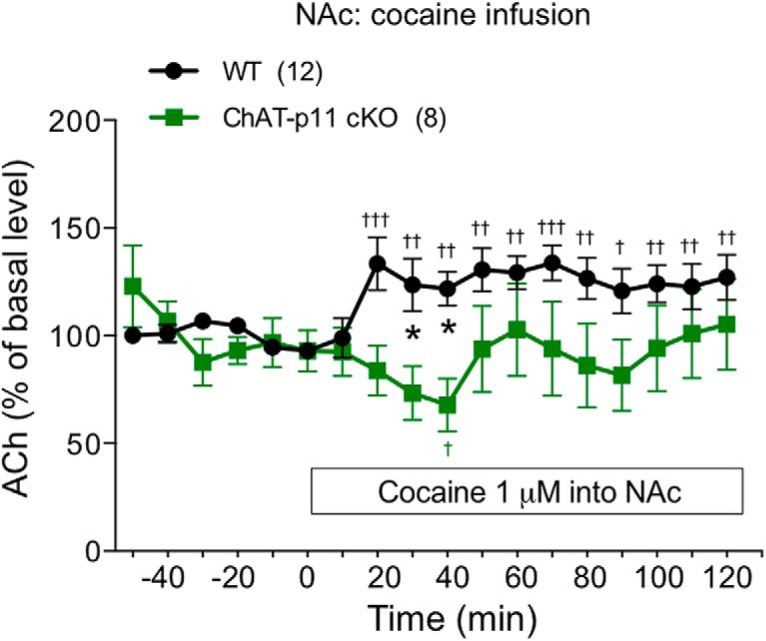
The ACh responses to cocaine infusion in the NAc of ChAT-p11 cKO mice. The extracellular levels of ACh in the NAc were measured with *in vivo* microdialysis after infusion of cocaine (1 µM) into the NAc of WT (ChAT-Cre^-/-^ p11^flox/flox^) and ChAT-p11 cKO (ChAT-Cre^+^ p11^flox/flox^) mice. The basal values for each group were obtained as the average of six stable baseline samples, and all values are calculated as a percentage of the basal values within the same group (100%). Data represent mean ± SEM. *p < 0.05 versus WT mice; two-way ANOVA and Bonferroni multiple comparison test. ^†^*p* < 0.05, ^††^*p* < 0.01, ^†††^*p* < 0.001 versus the basal levels of ACh in the same group. The number of mice is indicated in parentheses under each experimental condition.

Pharmacological analyses suggested that p11 in NAc CINs is required for the dopamine responses to rewarding stimuli presumably via mechanisms involving ACh release from CINs and activation of dopaminergic terminals by ACh. We therefore measured the extracellular levels of ACh after cocaine infusion in the NAc of WT and ChAT-p11 cKO mice (Fig. 5). Cocaine infusion (1 µM) into the NAc increased the levels of ACh to 130–140% of control in the NAc of WT mice, but failed to increase them in the NAc of ChAT-p11 cKO mice. These results confirm that cocaine induces the release of ACh from CINs and that p11 is essential for the cocaine-induced release of ACh. It is likely that the released ACh together with the inhibition of dopamine transporter by cocaine increases the extracellular levels of dopamine in the NAc.

### Effects of chemogenetic activation of NAc CINs on the dopamine responses to cocaine in ChAT-p11 cKO mice

**Figure 6.**
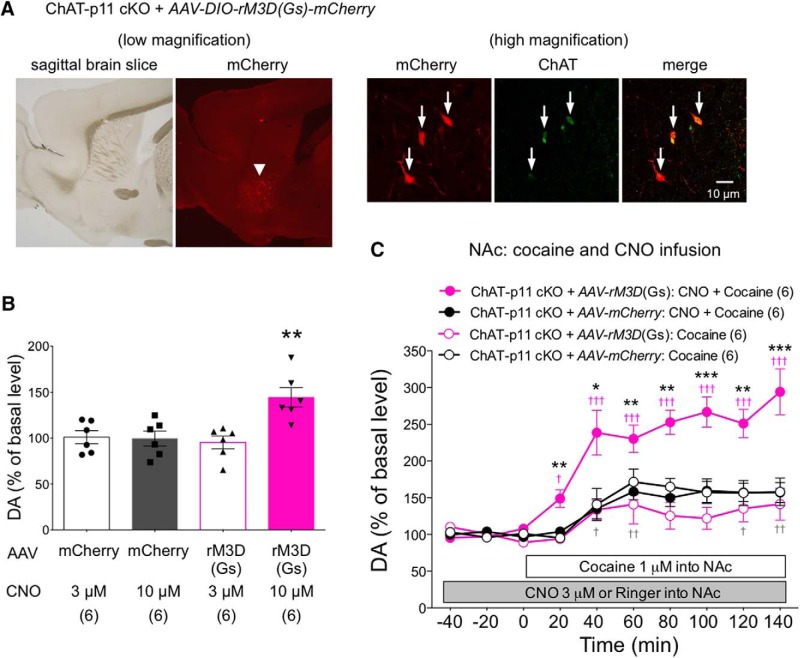
Activation of ChAT cells in the NAc using a chemogenetic technique restores the dopamine (DA) response in ChAT p11 cKO mice. ***A***, Immunohistochemical detection of mCherry (red) and ChAT (green) in the NAc of ChAT-p11 cKO mice injected with Gs-DREADD virus [*AAV-DIO-rM3D(Gs)-mCherry* (*AAV-rM3D(Gs)*)] into the NAc. In images with low magnification (left panel), mCherry-positive cells are aparsely dstributted in the NAc (arrow head). In images with high magnification (right panel), mCherry is expressed in ChAT-positive CINs. Arrows indicate ChAT-positive CINs expressing rM3D(Gs). ***B***, The effects of CNO infusion at 3 or 10 µM into the NAc on the extracellular levels of DA in the NAc of ChAT-p11 cKO mice injected with control [*AAV-DIO-mCherry* (*AAV-mCherry*)] or Gs-DREADD virus. The DA levels were determined as the average of those at 40, 60, and 80 min of CNO infusion. Data represent mean ± SEM. **p < 0.01; one-way ANOVA and Newman–Keuls multiple comparison test. ***C***, The effects of CNO infusion (3 µM) into the NAc on the cocaine-induced increases in DA in the NAc of ChAT-p11 cKO mice injected with control (*AAV-mCherry*) or Gs-DREADD virus. The basal values for each group were obtained as the average of three stable baseline samples, and all values are calculated as a percentage of the basal values within the same group (100%). Data represent mean ± SEM. *p < 0.05, **p < 0.01, ***p < 0.001 versus ChAT-p11 cKO mice with control virus injection; two-way ANOVA and Bonferroni multiple comparison test. ^†^*p* < 0.05, ^††^*p* < 0.01, ^†††^*p* < 0.001 versus the basal levels of DA in the same group. The number of mice is indicated in parentheses under each experimental condition.

Our studies using p11 KO and ChAT-p11 cKO mice with pharmacological and viral tools strongly suggested that cholinergic regulation of dopamine release is attenuated following deletion of p11 in NAc CINs. Next we investigated whether chemogenetic activation of NAc CINs may restore the attenuated dopamine responses to cocaine in ChAT-p11 cKO mice. Gs-DREADD (*AAV-DIO-rM3D(Gs)-mCherry*) or control (*AAV-DIO-mCherry*) virus was injected into the NAc of ChAT-p11 cKO mice. After four weeks of Gs-DREADD viral injection, mCherry was expressed in ChAT-positive large-sized neurons in the NAc (Fig. 6*A*), suggesting the expression of rM3D(Gs) in CINs. CNO was locally infused into the NAc via the microdialysis probe. CNO infusion of 3 µM did not affect the basal levels of dopamine in ChAT-p11 cKO mice with Gs-DREADD viral injection (Fig. 6*B*). CNO infusion at a higher concentration (10 µM) increased the average of dopamine levels at 40, 60, and 80 min of CNO infusion in the NAc of ChAT-p11 cKO mice with Gs-DREADD viral injection, but not with control viral injection. These results suggest that chemogenetic activation of CINs alone induces the release of dopamine in the NAc, only when a high concentration of CNO (10 µM) was infused.

We next evaluated the effects of chemogenetic activation of CINs on the dopamine responses to cocaine. After observing that CNO infusion (3 µM) for 140 min did not affect the basal levels of dopamine, cocaine infusion (1 µM) into NAc was started. Cocaine infusion together with CNO infusion (3 µM) induced the dopamine responses in the NAc of ChAT-p11 cKO mice with Gs-DREADD viral injection (Fig. 6*C*). Restoration of dopamine responses was not achieved in animals treated with Gs-DREADD plus cocaine without CNO or in animals treated with mCherry virus plus cocaine/CNO. These results suggest that activation of CINs is required for dopamine responses to rewarding stimuli in the NAc, and that p11 is essential for CIN activation.

### Effects of chemogenetic inhibition of NAc CINs on the dopamine responses to cocaine in control mice

**Figure 7.**
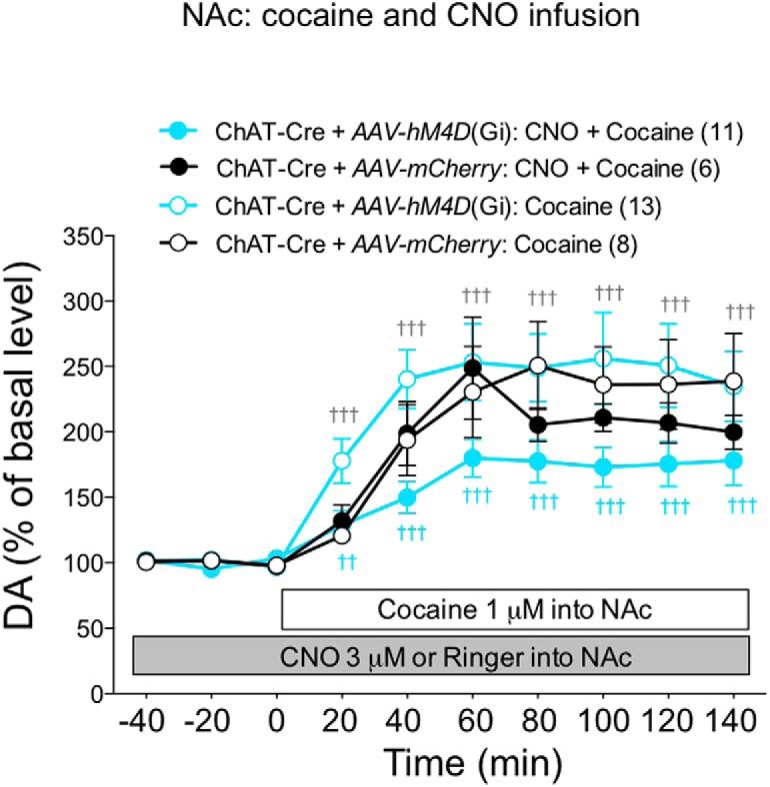
Inhibition of ChAT cells in the NAc using a chemogenetic technique suppresses the dopamine (DA) response in control mice. Gi-DREADD virus [*AAV-DIO-rM4D(Gi)-mCherry* (*AAV-rM4D(Gi)*)] or control virus [*AAV-DIO-mCherry* (*AAV-mCherry*)] was injected into the NAc of ChAT-Cre mice. The effects of CNO infusion (3 µM) into the NAc on the cocaine-induced increases in DA in the NAc were examined. The basal values for each group were obtained as the average of three stable baseline samples, and all values are calculated as a percentage of the basal values within the same group (100%). Data represent mean ± SEM. ^††^*p* < 0.01, ^†††^*p* < 0.001 versus the basal levels of DA in the same group. The number of mice is indicated in parentheses under each experimental condition.

We further investigated whether the inhibition of NAc CINs by Gi-DREADD could suppress the dopamine response to cocaine infusion in the NAc of ChAT-Cre mice injected with Gi-DREADD virus (*AAV-DIO-rM4D(Gi)-mCherry*) or control virus (*AAV-DIO-mCherry*) (Fig. 7). In ChAT-Cre mice expressing Gi-DREADD, CNO infusion (3 µM) into the NAc attenuated the dopamine response to cocaine infusion (1 µM). CNO infusion into the NAc of ChAT-Cre mice without Gi-DREADD expression did not affect the dopamine response to cocaine infusion.

## Discussion

In this study, we demonstrated that p11 expressed in CINs of the NAc is a critical regulator of the dopamine reward system. *In vivo* microdialysis analyses in constitutive p11 KO mice revealed that lack of p11 induced the attenuation of dopamine responses to rewarding stimuli including a drug of abuse and natural rewards. The attenuation of the dopamine responses in the mesolimbic (VTA-NAc) dopamine system, but not in the mesocortical (VTA-PFC) dopamine system, suggested the importance of p11 in the NAc. The dopamine responses were attenuated in ChAT-p11 cKO mice, and the attenuated responses were restored by the overexpression of p11 in NAc CINs, indicating the critical role of p11 in NAc CINs. Furthermore, lack of p11 in NAc CINs results in the attenuation of ACh release in response to cocaine and the subsequent decrease in nicotinic and muscarinic ACh receptor signaling at dopaminergic terminals, leading to the suppressed dopamine responses to cocaine and possibly other rewarding stimuli. The function of p11 in CINs was confirmed by the chemogenetic studies: CIN activation by Gs-DREADD restored the dopamine responses in ChAT-p11 cKO mice, whereas CIN inhibition by Gi-DREADD suppressed the dopamine response in control (ChAT-Cre) mice. Thus, p11 in NAc CINs is required for the dopamine response of the mesolimbic rewarding system. These findings provide insights into the neural mechanisms of anhedonia in depression.

### Selective regulation of the mesolimbic dopamine pathway by p11

p11 regulates the dopamine response to rewarding stimuli in the mesolimbic dopamine pathway, but not in the mesocortical dopamine pathway. Selective regulation of the mesolimbic dopamine pathway is enabled by action of p11 in CINs of the NAc. PFC receives cholinergic innervation from the basal forebrain (Ballinger et al., 2016), and ChAT cells in the basal forebrain also express p11 (Milosevic et al., 2017). Although p11 in ChAT cells of the basal forebrain is deleted in ChAT-p11 cKO mice, the deletion of p11 did not alter the dopamine response in the mesocortical dopamine pathway. A possible role for p11 in the cholinergic neurons of the basal forebrain needs to be explored in other brain functions such as cognition (Ballinger et al., 2016). Furthermore, the fact that the lack of p11 in VTA dopamine neurons of p11 null mice did not affect the dopamine responses in the mesocortical dopamine pathway suggests a limited role for p11 in regulating the activity of the dopaminergic neurons of the VTA. In fact, this interpretation is consistent with the low expression of p11 in the VTA (Milosevic et al., 2017). Thus, p11 in CINs is a critical regulator of the dopamine response to rewarding stimuli in the mesolimbic dopamine pathway.

### Functional role of p11 in the regulation of CIN activity and ACh release in the NAc

Cholinergic tone in the mesolimbic dopamine system plays an important role in behavioral responses to psychostimulants and natural reward (Hoebel et al., 2007; Williams and Adinoff, 2008). It has been demonstrated that silencing CIN activity induces depression-like behaviors and that p11 in NAc CINs shows antidepressant effects (Warner-Schmidt et al., 2012). Our findings indicate that, in the NAc, activation of CINs and the subsequent release of ACh are required for dopamine responses to rewarding stimuli, and that p11 is essential for CIN activation in response to reward. It is likely that ACh released from CINs in a p11-dependent manner activates the dopamine release machinery via activation of α4β2 nAChRs (Wonnacott et al., 2000; Hamada et al., 2004) and M5 muscarinic receptors (Bendor et al., 2010; Kuroiwa et al., 2012) at dopaminergic axon terminals, leading to the enhancement of the increase in extracellular dopamine induced by cocaine, a dopamine reuptake inhibitor. Furthermore, p11-dependent activation of CINs and ACh release seems to be optimal to enhance the dopamine reward probability, because the inverted U-shape threshold model suggests that activation of CINs above a certain threshold reduces it (Grasing, 2016). This is in line with a previous report that basal ACh release is unchanged in ChAT-p11 cKO mice (Virk et al., 2016). Interaction of p11 with its binding proteins such as the 5-HT_1B_ receptor, 5-HT_4_ receptor and mGluR5 are required for antidepressant action (Svenningsson et al., 2006; Warner-Schmidt et al., 2009; Lee et al., 2015), but the precise p11-mediated mechanisms for CIN activation were unknown. The interaction of p11 with 5-HT_1B_ receptors in CINs may induce the inhibition of CIN activity (Virk et al., 2016), but this mechanism cannot explain our findings. Future studies should determine the molecular mechanisms by which p11 and presumably the p11 complex may activate CINs.

### Role of p11 in CINs of the NAc in anhedonic behaviors of depression

Anhedonia is a core symptom of depression. It has been shown that anhedonia is associated with a deficit in the mesolimbic dopamine circuit (Der-Avakian and Markou, 2012; Russo and Nestler, 2013). Current antidepressants are relatively ineffective for treating anhedonia (Craske et al., 2016), probably because depressive patients are treated with antidepressants primarily acting on 5-HT and/or noradrenaline transmission (Dunlop and Nemeroff, 2007). To develop a new type of antidepressant effective for anhedonia, it is extremely important to elucidate the mechanism by which the mesolimbic dopamine reward circuit is dysregulated in depression. In this study, we clearly demonstrated that p11 in NAc CINs is a critical regulator of the mesolimbic dopamine response to rewarding stimuli. The findings suggest that p11, which is required for activation of CINs and the ACh release in response to rewarding stimuli, plays a pivotal role in the pathophysiology of anhedonia in depression (Svenningsson et al., 2006).

Deletion of p11 in ChAT cells, p11 knock-down in the NAc or silencing NAc CINs induces anhedonic behavior, and overexpression of p11 in NAc CINs reverses anhedonic behavior in constitutive p11 KO mice (Alexander et al., 2010; Warner-Schmidt et al., 2012). In addition, p11 expression in the NAc is reduced in depressed patients (Svenningsson et al., 2006; Alexander et al., 2010). Thus, the reduction of p11 expression in NAc CINs is tightly associated with anhedonia as well as other depression-like symptoms of behavioral despair. Therapeutic strategies that increase the expression of p11 and the signaling of the p11 complex in NAc CINs may have impact on current antidepressant treatment.

In conclusion, p11 is a critical regulator of CIN activity as measured by the dopamine response of the mesolimbic dopamine pathway to rewarding stimuli. p11 is required for reward-mediated NAc CIN activation and induction in ACh release, resulting in the enhancement of dopamine release. To improve therapeutic efficacy of antidepressants for anhedonia, a new type of antidepressant directly or indirectly acting on the mesolimbic dopamine pathway needs to be developed. For this purpose, p11 and its complex in the NAc CINs may be good therapeutic targets.

## Synthesis

Reviewing Editor: Karen Szumlinski, University of California at Santa Barbara Decisions are customarily a result of the Reviewing Editor and the peer reviewers coming together and discussing their recommendations until a consensus is reached. When revisions are invited, a fact-based synthesis statement explaining their decision and outlining what is needed to prepare a revision will be listed below. The following reviewer(s) agreed to reveal their identity: Emmanuel Valjent, Denis Herve.

Following consultation with two experts reviewers in the field of dopamine neurobiology, we have reached the decision of Revise-Editor Review only. Both reviewers found your article to be of excellent quality. Both of the reviewers had some minor suggestions for improvement that should be included in a revision. I have copied their comments below. I look forward to receiving the revision.

Reviewer 1

Excellent study providing a clear demonstration of the contribution of p11 expressed by NAc cholinergic interneurons in the control of DA release induced by rewarding stimuli.

By combining microdialysis with pharmacological and chemogenetic approaches in p11 cKO mice, the authors demonstrated the critical role of p11 expressed by NAc cholinergic interneurons in controlling DA release induced by rewarding stimuli. The results are clear and convincing. The quality of the data is remarkable and the manuscript is concise and well written.

I only have minor requests.

- The authors should show the histological control ie localization of the implantation of the microdialysis probes.

- It would be also informative to show lower magnification in order to appreciate the extent of the viral injection (Figure 4a,b and 6)

- I strongly encourage the authors to submit a visual abstract

Reviewer 2

This study is focusing p11 a protein whose alteration has been clearly implicated in depression. Previous work had shown that alteration of the p11 protein in the cholinergic neurons of the nucleus accumbens led to depressive-like behaviors in mice, including anhedonia, which is one of the symptoms the least responsive to current treatments for depression. The present study shows that the selective absence of p11 in cholinergic neurons alters the dopamine release in response to rewarding stimuli. This effect is a plausible mechanism for the resulting anhedonia. In addition, the restoration of p11 expression in cholinergic neurons or chemogenetic stimulation of cholinergic neurons induce the recovery of normal responses. This study represents a noticeable advance in our understanding of the role of p11 in depression.

The manuscript entitled “p11 in cholinergic interneurons of the nucleus accumbens is essential for dopamine responses to rewarding stimuli” is of excellent quality. It reports microdialysis results on the dopamine and acetylcholine release in response to cocaine and reinforcing stimuli. By complementary and well-designed approaches, the experiments clearly show the role of p11 in the cholinergic neurons of the nucleus accumbens on the dopamine responses. The results are clear and correctly analyzed notably by accurate statistical methods. The conclusions are completely supported by the data presented and have a significant scientific impact considering the involvement of p11 in depression, including the symptoms of anhedonia. The molecular mechanisms underlying the role of p11 in the activity of cholinergic neurons and their responses to rewarding stimuli are not investigated, but are clearly beyond the objectives of the study.

I have only a few minor remarks about the manuscript.

1- The authors show that stimulation of nicotinic or muscarinic receptors has effects on p11-dependent dopamine release. They could discuss how these receptors could regulate dopamine release in the nucleus accumbens.

2- The infusion of 1μM of cocaine into the nucleus accumbens is considered as a rewarding stimulus in the manuscript. What literature elements support this statement?

3- The description of the different mouse lines and their crosses should be more developed in the Material and Methods part.

4- The definition of the basal release level should be more clearly defined in the figure legends.

5- An illustration of the location of the microdialysis cannulas in the nucleus accumbens would be interesting to show.

6- Finally, some elements of the literature suggest that cholinergic neurons have an inhibiting role on behavioral responses to cocaine and others a stimulating role. The authors could discuss their data in relation to these studies, which seem divergent at first glance.
